# Targeting Hyperoxia‐Induced Cellular Senescence in Developing Human Airway Cells: Senomorphics Versus Senolytics Versus Antioxidants

**DOI:** 10.1111/acel.70538

**Published:** 2026-05-08

**Authors:** Maunick Lefin Koloko Ngassie, Li Y. Drake, Yi Zhu, Yamillie Ortiz, Daniel A. Pfeffer‐Kleemann, Michael A. Thompson, Samantha K. Hamrick, Christina M. Pabelick, Y. S. Prakash

**Affiliations:** ^1^ Department of Anesthesiology and Perioperative Medicine Rochester Minnesota USA; ^2^ Department of Physiology and Biomedical Engineering Mayo Clinic Rochester Minnesota USA

**Keywords:** asthma, bronchopulmonary dysplasia, Dasatinib, Fucoidan, lung development, MitoQ, oxygen, Quercetin

## Abstract

Supplemental oxygen (hyperoxia), often provided to premature infants, can disrupt lung growth and contribute to development of neonatal and pediatric lung diseases, necessitating understanding of underlying mechanisms. We previously showed that even moderate hyperoxia (< 60% O_2_) induces detrimental cellular senescence in 18–22 weeks human fetal airway smooth muscle (fASM), a key cell type in airway contractility and remodeling. In this study, we examined the ability of senotherapeutics Fucoidan and Dasatinib (D) + Quercetin (Q) (D + Q) to mitigate adverse effects of hyperoxia and assessed the preventive effect of mitochondrial antioxidants (MitoQ). fASM cells exposed to normoxia (21% O_2_) or hyperoxia (50% O_2_) were treated with Fucoidan [100 μg/mL], D + Q [250 nM + 375 nM] or MitoQ [100 nM] and assessed for senescence markers, senescence‐associated secretory profile (SASP), extracellular matrix (ECM) deposition, and cell viability. Our results showed that moderate hyperoxia increases senescence markers and SASP. Fucoidan decreased p21 expression and inhibited SASP release without inducing cell death, while D + Q decreased β‐galactosidase (β‐Gal) activity, p21 and plasminogen activator inhibitor‐1 (PAI‐1) expression, and caused cell death without affecting SASP in hyperoxia‐induced senescent fASM. MitoQ prevented increases in senescence markers and SASP in hyperoxia‐exposed fASM. These findings demonstrate distinct and viable strategies to counter hyperoxia‐induced fASM senescence. Fucoidan (senomorphic), D + Q (senolytic), and MitoQ (prophylactic antioxidant) represent promising, mechanistically different approaches to alleviate detrimental effects of moderate hyperoxia in developing airways towards limiting perinatal lung disease.

## Graphical abstract

Supplemental oxygen is critical for premature infants. While initially beneficial, even moderate hyperoxia (< 60% O_2_) induces detrimental cellular senescence in developing airways with long‐lasting consequences. Senotherapeutics such as Fucoidan, Dasatinib (D) + Quercetin (D + Q), and mitochondrial‐targeted antioxidant MitoQ may be beneficial in mitigating detrimental effects of hyperoxia.

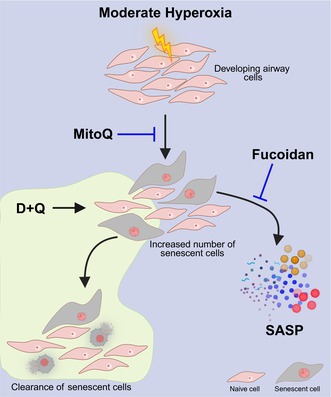

## 1 Introduction

Approximately 11% of all infants worldwide are born preterm (< 37 weeks gestation; WHO report, 2012). Premature infants are often administered supplemental O_2_ as a life‐supporting therapy to maintain oxygenation (Martin and Fanaroff 2013). Recognizing that very high O_2_ promotes development of bronchopulmonary dysplasia (BPD), clinical practice has trended towards moderate O_2_ (< 60% O_2_). However, even moderate O_2_ in prematurity is associated with increased risk for subsequent development of bronchial disease involving inflammation, tissue remodeling and airway hyperresponsiveness (AHR) (Been et al. 2014; Doyle and Anderson 2010; Holditch‐Davis et al. 2008; Martin and Fanaroff 2013). AHR involves increased contractility of airway smooth muscle (ASM) in response to bronchoconstrictor agonists (e.g., acetylcholine, histamine) (Prakash 2016). Remodeling involves alterations in the epithelial layer, hypertrophy and hyperplasia of ASM, and contributions of ASM and fibroblasts to increased extracellular matrix (ECM) deposition (fibrosis) (Cockcroft and Davis 2006; Prakash et al. 2017; Zhang, Bartman, et al. 2023). However, the mechanisms by which hyperoxia leads to later development of bronchial airway disease are still being investigated and represent an unmet need in preventative and/or therapeutic strategies for preterm infants and children.

Emerging evidence suggests that cellular senescence contributes to the pathology of bronchial airway diseases, such as pulmonary fibrosis, chronic obstructive pulmonary disease (COPD), bronchiectasis, and asthma (Aghali et al. 2022; Antony and Thannickal 2018; Schafer et al. 2017). Cellular senescence is generally induced by stress or DNA damage and characterized by irreversible cell cycle arrest and production of senescence‐associated secretory phenotype (SASP) proteins, including proinflammatory cytokines and chemokines, growth factors, and matrix remodeling factors (Zhang, Pitcher, et al. 2023). Although the senescence and SASP profiles are cell type‐ and context‐dependent (Childs et al. 2018; Parikh, Wicher, et al. 2019), some classic markers include higher levels of DNA damage, β‐galactosidase activity, phospho‐p53, p21, p16, and anti‐apoptotic factors, along with decreased cell proliferation (Hayflick and Moorhead 1961; Herbig et al. 2004). Recent studies suggest that senescence contributes to hyperoxia‐induced pathology in developing lungs. For example, we demonstrated that moderate hyperoxia induces senescence in human fetal airway smooth muscle (fASM) cells and fetal lung fibroblasts (Koloko Ngassie et al. 2025; Parikh, Britt Jr., et al. 2019; You et al. 2019). Additionally, SASP proteins including interleukin 1 alpha (IL1α), IL‐6, IL‐8, and plasminogen activator inhibitor‐1 (PAI‐1) were upregulated in supernatants or cell lysates of hyperoxia‐induced senescent fASM. Analysis of human neonatal lung autopsy specimens revealed that O_2_ supplementation elevates senescence‐associated marker expression in the ASM layer (Parikh, Britt Jr., et al. 2019), highlighting clinical relevance. Therefore, developing therapeutic strategies targeting senescent cells may offer beneficial outcomes for premature infants requiring O_2_ supplementation.

Currently, there are two approaches to modulating senescence: killing senescent cells using senolytics or inhibiting SASP release using senomorphs. Previous studies showed that senescent cells could be cleared using the senolytic combination of the tyrosine kinase inhibitor Dasatinib and the flavonoid Quercetin (D + Q) (Zhu et al. 2015). Senomorphs have shown their efficacy in mitigating SASP secretion. For example, Fucoidan, a seaweed‐derived compound with anti‐inflammatory potential, was recently shown to serve as senolytic as well as senomorphic (Ahmad et al. 2021; Robbins et al. 2025). Fucoidan can affect several cellular pathways including nuclear factor k‐light‐chain‐enhancer of activated B cells (NF‐kB), mitogen‐activated protein kinases (MAPKs), transforming growth factor‐beta (TGF‐β), and nuclear factor erythroid 2‐related factor 2 (NRF‐2) (Zayed et al. 2023). These pathways are involved in senescence, either as inducers (MAPKs, TGF‐β, and NF‐kB) or inhibitors (NRF‐2) (Kumari and Jat 2021; Yuan et al. 2021). Besides senolytics and senomorphs, given the fact that hyperoxia can induce oxidant stress, there is interest in antioxidants to alleviate hyperoxia effects. Indeed, even moderate hyperoxia promotes links between mitochondrial reactive oxidative species (ROS) and cellular senescence (Koloko Ngassie et al. 2025). Accordingly, there is interest in whether mitochondria‐targeted ROS inhibitors could be used as preventive measures.

While previous data underline a link between O_2_ and senescence, a major limitation in the field has been the characterization of senescent cells per se and their sub‐populations, due to the lack of robust markers. Since many senescent markers are also expressed in non‐senescent cells (Suryadevara et al. 2024), precise identification of senescent cells requires multiparametric phenotyping. The relevance of senescent cell phenotyping lies in the fact that senescence also has beneficial effects in growth and repair (Parikh, Wicher, et al. 2019) (particularly relevant in the developing and perinatal lung) but can become detrimental when senescent cell numbers exceed immune clearance (Prata et al. 2018), and a greater proportion of such cells secrete pro‐inflammatory, pro‐fibrotic SASP with paracrine effects on surrounding naïve tissues (Tchkonia et al. 2013) (a particular issue in developing lung with immature immune systems). Accordingly, it becomes important to identify senescent cell sub‐populations in the context of exposures to understand the cost‐benefits of interfering with senescence.

In this study, we hypothesized that senotherapeutics and antioxidants are potential approaches to mitigate detrimental effects of hyperoxia‐induced senescent fASM. Our aims were to identify therapeutic approaches that can (1) eliminate hyperoxia‐induced senescent fASM, (2) inhibit secretion of hyperoxia‐induced SASP, or (3) prevent hyperoxia‐induced senescence in fASM via the use of mitochondria‐targeted antioxidant. Furthermore, we used this in vitro model to explore a robust protocol to phenotype senescence in developing airway cells.

## 2 Materials and Methods

### 2.1 Isolation of Human Fetal ASM

Human tracheobronchial tissues were enzymatically isolated from anonymized 18–22 week gestational age fetuses following demise (considered exempt per Mayo Clinic Institutional Review Board (IRB)). The detailed procedure for fASM isolation has been described previously (Koloko Ngassie et al. 2025). Cells from passages 1–7 (typically < 3) of subculture were used for experiments. ASM phenotype was regularly verified by westerns or cellular staining for smooth muscle markers including smooth muscle myosin (Supporting Information S1, Figure S1B) and by positive robust intracellular calcium responses of the cells to bronchoconstrictor agonists (acetylcholine and histamine) (Supporting Information S1, Figure S2) (Britt Jr. et al. 2015; Hartman et al. 2012). Additionally, fASM were verified to negatively stain for fibroblast specific protein (FSP) (Supporting Information S1, Figure S1A). Each sample represents cells isolated from an individual lung (biological replicates).

### 2.2 Cell Culture and Treatments

For culture of primary fASMs, Dulbecco's Modified Eagle's Medium/Ham's Nutrient Mixture F12 (DMEM/F‐12) with low glucose 1 g/L (R&D Systems, Lot #M23850) supplemented with 10% fetal bovine serum (FBS) (R&D Systems, Lot #G22143) and 1% antibiotics and antimycotic (AA) (Gibco, Lot #2441424) was used and cells incubated at 37°C and 5% CO_2_. Based on our previous model of hyperoxia‐induced cellular senescence in fASM (Koloko Ngassie et al. 2025; Parikh, Britt Jr., et al. 2019). 25,000 cells were seeded per well in a 12‐well plate. After 24 h, cells were growth‐arrested in 0.5% FBS media for another 24 h and then exposed for seven consecutive days to normoxia (21% O_2_) or hyperoxia (50% O_2_). Subsequently, cells were treated with Fucoidan or D + Q (Supporting Information S1, Figure S3) in a normoxic environment. This model was intended to mimic early oxygen exposure in the NICU when senescence would be induced, followed by potential treatment in subsequent normoxic environment when reduction or removal of senescence cells would be needed.

#### 2.2.1 Fucoidan and Dasatinib + Quercetin (D + Q) Treatments

Detrimental effects of senescence can potentially be blunted via senotherapeutics (senolytics and senomorphs). Fucoidan has been described as both senolytic or senomorph depending on its source (Robbins et al. 2025) and has shown to be anti‐inflammatory at least in vitro (Ahmad et al. 2021). In pilot experiments we tested three different concentrations of Fucoidan (Selleckchem, cat: E0365; 25, 50 and 100 μg/mL) on p21 expression in hyperoxia‐exposed fASM (Supporting Information S1, Figure S4). For the final experiments, cells were treated with growth medium (control) or Fucoidan [100 μg/mL] for 24 h (day 8) in normoxia.

In parallel, we treated cells with the widely‐used senolytic cocktail D + Q. The concentration of D + Q was selected based on our previous study (Parikh, Britt Jr., et al. 2019). After 7 days of exposure to normoxia or hyperoxia, cells were treated with 0.05% DMSO (vehicle) or D + Q [250 nM + 375 nM] (Dasatinib, Sigma, cat: CDS023389; Quercetin, Sigma, cat: Q4951) for 24 h (day 8) in normoxia. Samples were replaced with fresh growth medium and cells incubated for an additional 24 h (day 9). Cell lysates and supernatants were harvested on day 8 and 9 and were analyzed for senescence markers and SASP proteins, respectively.

#### 2.2.2 MitoQ Treatment

Primary fASM cells were seeded in 10 cm petri dishes at 1.2 × 10^5^ cells per dish. After 24 h, cells were growth‐arrested in 0.5% FBS media for another 24 h and were then cultured in either normoxia or hyperoxia with 5% CO_2_ at 37°C for 7 days. To investigate the role of mitochondrial ROS in senescence and SASP production, MitoQ [100 nM] (Selleckchem, cat: S8978) was added to the culture for 7 days, as established in our previous study (Koloko Ngassie et al. 2025). Cells were harvested via trypsinization at day 7, followed by CyTOF antibody staining. This was considered a preventative strategy.

### 2.3 Proliferation Assay

3,000 cells/well were seeded in 96‐well black/clear bottom plates and cultured for 7 days in normoxia or hyperoxia. Then, cells were examined for cell proliferation using the CyQUANT NF Cell Proliferation Assay kit (C35006; Invitrogen, Carlsbad, CA). Growth medium was discarded and 50 μL of 1× dye binding solution containing 1× Hanks' balanced salt solution, 1× dye delivery reagent, and 1× CyQUANT NF dye reagent were added per well. Subsequently, the plate was placed back in the incubator for 1 h at 37°C and 5% CO_2_ to enable the binding of the dye to the DNA. Finally, the fluorescent signal was measured with excitation at 485 nm and emission at 530 nm using a FlexStation 3 (Molecular Devices; San Jose, CA).

### 2.4 ECM Deposition Assay

To quantify the deposition of specific ECM proteins such as collagen type I (COL1), COL3 or fibronectin (FN1), cells used for the proliferation assay were washed once with Dulbecco's Phosphate Buffered Saline (DPBS) and lysed with 100 μL/well of ammonium hydroxide (NH_4_OH) [16 mM/L]. The plate was incubated for 30–40 min at room temperature (RT) on a rocker until complete cell lysis or lifting was microscopically confirmed. Then the plate was washed three times with DPBS, followed by blocking of the unspecific binding sites by adding 100 μL/well Intercept Blocking Buffer (Lot: 240826; LI‐COR Biosciences, Lincoln, NE) and incubating the plate for 1 h at RT with gentle shaking. After 50 μL/well of primary antibodies: COL1 (polyclonal rabbit, ab34710, 1:100, abcam), COL3 (polyclonal rabbit, ab7778, 1:100, abcam) and FN1 (polyclonal rabbit, ab2413, 1:1000, abcam) were added and the plate incubated overnight at 4°C on a rocker. Next day, the plate was washed five times each 5 min with Tris‐buffered saline with 0.1% Tween 20 (TBST). Secondary antibodies: IRDye 800CW Goat anti‐Rabbit IgG (cat: 926‐32211, 1:400, LI‐COR Biosciences, Lincoln, NE) were added and the plate incubated for 1 h at RT (in the dark) on a rocker. Then, the plate was washed five times each 5 min with TBST and dried in the dark. Finally, ECM fluorescence mean intensities were acquired using LI‐COR ODYSSEY M (LI‐COR Biosciences, Lincoln, NE) and Empiria Studio 2.2 software (LI‐COR Biosciences, Lincoln, NE). The obtained mean intensities were normalized to cell number.

### 2.5 Cell Viability Assay

To assess cell viability, 3000 cells were plated in 96‐well plates and treated as described in section *Fucoidan and Dasatinib + Quercetin (D + Q) treatments*. At day 8, culture medium was removed, and cells were washed once with DPBS. Cells were fixed with 4% paraformaldehyde (PFA) in PBS for 15 min at RT. After fixation, cells were washed twice with distilled water and allowed to airdry at RT for 30 min. Then, cells were stained with 0.1% crystal violet staining solution and incubated for 30 min at RT in the dark. Cells were washed several times with distilled water until all excess dye was removed. Plates were allowed to airdry overnight. Representative images were taken using LI‐COR ODYSSEY M (LI‐COR Biosciences, Lincoln, NE) imaging system. Finally, Crystal Violet stain was solubilized with a 1% SDS solution and incubated for 30 min at RT. Absorbance (optical density, OD) was measured at a 590 nm wavelength using a FlexStation 3 (Molecular Devices; San Jose, CA).

### 2.6 Protein Analysis

A capillary‐based electrophoresis system (ProteinSimple JESS, Santa Clara, CA) was used to quantify protein expression as described previously (Koloko Ngassie et al. 2025). Samples were prepared following the manufacturer's protocol. Expression levels of senescence markers including p21, plasminogen activator inhibitor‐1 (PAI‐1) and lamin B1 (LMNB1) were determined using the following primary antibodies: Anti‐p21 antibody (monoclonal rabbit, ab109520, 1:50, abcam), PAI‐1 (monoclonal rabbit, 49536S, 1:25, Cell Signaling Technology) and anti‐Lamin B1 antibody (monoclonal rabbit, ab133741, 1:50, abcam), respectively. The anti‐rabbit secondary HRP antibody (cat: 042‐206) was from ProteinSimple (San Jose, California, USA). The Simple Western Total Protein Assay (DM‐TP01) was used to normalize protein expression. Data are reported as protein relative expression to total protein. Images of the capillary showing the specificity of the primary antibodies used are available in Supporting Information S2 (Figures S1–S3).

### 2.7 Evaluation of β‐Galactosidase Activation

In 96‐well black/clear bottom plate, 3000 fASM were seeded and cultured as described in section *Cell culture and treatments*. Cells were stained at day 0, 1, 3, 7, and 8. Before starting the staining procedure, cells were fixed using 4% paraformaldehyde (PFA) solution for 10 min at RT in the dark. Then, cells were washed with DPBS and 100 μL of 1× CellEvent Senescence Green Probe (staining solution) prepared with 37°C pre‐warmed CellEvent Senescence Buffer was added onto cells. The plate was sealed with parafilm and incubated for 2 h at 37°C without CO_2_. The staining solution was then discarded and the plate washed three times with 100 μL of DPBS per well. Finally, 100 μL of DPBS was added to each well and cells were imaged using Alexa Fluor 488/FITC filter set, with a Nikon fluorescence microscope and a dry ×20 objective. The mean intensities were normalized to cell number. For each primary cell, technical duplicates were performed and the mean intensities presented are averages of the mean intensities from 402 to 1118 cells per condition.

### 2.8 Analysis of SASP Profiles

Cell supernatants from Fucoidan and D + Q treatments were analyzed for cytokines, chemokines, and growth factors secretion. Samples were analyzed by Eve Technologies Corporation (Calgary, Alberta, Canada) using Luminex xMAP. Two panels, including the Human Cytokine/Chemokine 96‐Plex Discovery Assay Array (HD96) and Human Cytokine/Chemokine 71‐Plex Discovery Assay Array (HD71), were used for the analysis of supernatants from day 8 and day 9, respectively.

### 2.9 Senescent Cell Identification by Mass Cytometry by Time‐Of‐Flight (CyTOF)

CyTOF was employed to establish single‐cell protein profiling of normoxia and hyperoxia‐treated fASM, and assess their response to MitoQ treatment. We constructed and validated a CyTOF antibody panel to identify senescent fASM cells, including antibodies recognizing p16, p21, p53, PS6K, phospho‐nuclear factor kappa B (p‐NF‐κB) p65, C‐X‐C motif chemokine ligand (CXCL)1, IL‐1α, IL‐1β, matrix metalloproteinase (MMP)2, MMP9, Activin A, and Ki67 (Table 1). These markers were chosen based on the literature and our own studies (Parikh, Wicher, et al. 2019; You et al. 2019) and were intended to represent a broad swath of proteins that are thought to be relevant to senescence. Furthermore, this approach allowed us to determine whether senescent cells with a pro‐inflammatory, potentially detrimental SASP vs. those considered “benign” (see discussion) were present in our groups.

**TABLE 1:** CyTOF antibodies.

Target	Company	Catalog	Metal label
p16/CDKN2A	Biolegend	675602	151Eu
p16	abcam	ab54210	173Yb
p21/CDKN1A	Standard BioTools	3159026A	159Tb
p53	Standard BioTools	3143018A	143Nd
p53	Standard BioTools	3150024A	150Nd
Ki67	Standard BioTools	3161007B	161Dy
p‐NF‐κB p65 [S529]	Standard BioTools	3166006A	166Er
PS6K	R&D system	MAB8962	144Nd
Activin A	R&D system	AF338	153Eu
IL‐1α	Biolegend	500102	176Yb
IL‐1β	abcam	ab2105	160Gd
CXCL1	R&D system	MAB275	141Pr
MMP2	abcam	ab271866	165Ho
MMP9	abcam	ab119906	169Tm

Each antibody was labeled with a unique metal tag. All antibodies were validated for CyTOF by the Mayo Clinic CyTOF Core Laboratory and by us. Due to potential concerns regarding antibody specificity for human p16 and p53, we used two antibodies from different vendors for each marker. Cells were intracellularly stained with the cocktail of 14 CyTOF antibodies depicted in Table 1. The staining was carried out following Maxpar Nuclear Antigen Staining protocol (Standard BioTools). Briefly, harvested fASM cells were resuspended in 1 mL of Nuclear Antigen Staining Buffer (Standard BioTools, Cat# 201063) and incubated for 30 min. Cells were then washed twice with Nuclear Antigen Staining Perm and resuspended in 50 μL of this buffer. An antibody cocktail of the entire phenotyping panel (Table 1) was prepared as a master mix and then added to the cells in 50 μL of volume. After 30 min of incubation at room temperature, cells were washed twice with Nuclear Antigen Staining Perm and then fixed for 10 min with 1.6% PFA in PBS. Following fixation and wash, cells were resuspended in 1 mL Cell‐ID Intercalator‐Ir‐125 μM (Standard BioTools, Cat# 201192A. 1:2500 dilution in Maxpar Fix and Perm Buffer) and incubated overnight at 4°C. Cells were subsequently centrifuged at 800× *g* for 5 min, resuspended in 90% FBS + 10% DMSO, and kept frozen at −80°C until data acquisition.

On the day of acquisition, cells were thawed, washed twice with Cell Acquisition Solution Plus (Standard BioTools), resuspended in 10% EQ four Element Calibration Beads (Standard BioTools) in Cell Acquisition Solution Plus, and filtered into a 5 mL strainer cap tube (Cat # 352235, Falcon) before loading onto a Helios CyTOF system (Standard BioTools). Data were acquired at a rate of 300 events per second, saved as FCS files, and normalized using the manufacturer's CyTOF software. Using Cytobank software, acquired CyTOF data were cleaned up by gating on live single cells and then visualized using the t‐Distributed Stochastic Neighbor Embedding (t‐SNE) algorithm. Flow Self‐Organizing Map (FlowSOM) then clustered these cells based on their marker expression patterns followed by a hierarchical meta‐clustering step to merge related clusters. The relative marker expression intensities per cluster were visualized by a heatmap. Quantified values were plotted using GraphPad Prism 10.0.0 (153).

### 2.10 Statistical Analyses

Data were analyzed using GraphPad Prism 10.0.0 (153) (GraphPad, San Diego, CA). For statistical analyses different tests were applied according to the experiment design and the research question. The statistical methods used to analyze each set of data are highlighted in the figure legends. A *p*‐value < 0.05 was considered statistically significant.

## 3 Results

### 3.1 Hyperoxia Increases β‐Galactosidase Activity in fASM

**FIGURE 1:**
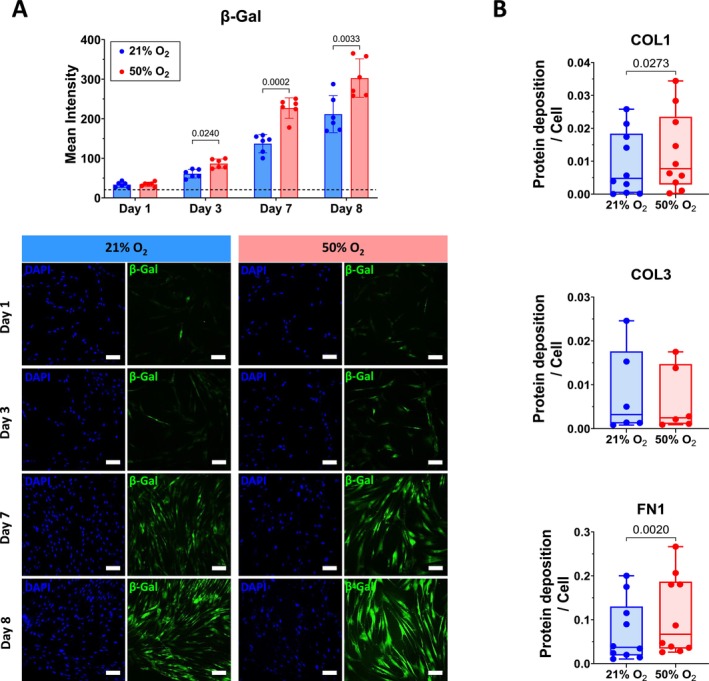
Hyperoxia increases senescence‐associated β‐Galactosidase (β‐Gal) activity and extracellular matrix (ECM) deposition in human fetal airway smooth muscle (fASM). (A) Cells were cultured under normoxic (21% O_2_) or hyperoxic (50% O_2_) condition for 8 days and β‐Gal activity was examined at different timepoints (day 0, 1, 3 7, and 8) using the CellEvent Senescence Green Detection Kit. Images were captured using Alexa Fluor 488/FITC filter set, with a Nikon fluorescence microscope and a dry ×20 objective. Technical duplicates were performed for each primary cell line and the mean intensities presented as the average from 418 to 873 cells per condition. (B) Cells cultured for 7 days in normoxia or hyperoxia were lysed, the deposited ECM stained for COL1, COL3 and FN1, and the acquired mean intensities were normalized to cell number. Hyperoxia increased the deposition of COL1 and FN1. For statistical analysis, 2‐way ANOVA with Šídák's multiple comparisons test (A) or Wilcoxon matched‐paired signed rank test (B) was applied. Data are presented as mean ± SD or box plot of *n* = 6–10 cell lines per group. *p* value < 0.05 was considered statistically significant. The black dotted line represents the level of β‐Gal activity at day 0. COL1, Collagen type I; COL3, collagen type III; FN1, fibronectin; SD, standard deviation. Scale bar: 100 μM.

Senescence‐associated β‐galactosidase (SA‐β‐Gal) activity is considered a “gold standard” marker for identification of senescent cells. We previously showed that moderate hyperoxia (40% O_2_) increased the percentage of β‐Gal‐positive fASM cells compared to normoxia (Parikh, Britt Jr., et al. 2019). In this study, using CellEvent Senescence Green Detection Kit, we performed time‐dependent activity of β‐Gal over 8 days at different time points (day 0, 1, 3, 7, and 8) in normoxia and hyperoxia (50% O_2_). From day 3 to day 8, significant increases were observed in β‐Gal activity in fASM exposed to hyperoxia compared to normoxia (Figure 1A).

### 3.2 Hyperoxia Increases ECM Deposition

Senescent cells are often characterized by their secretory profile, that is, SASP. ECM proteins are considered part of SASP and may contribute to the detrimental effect of SASP. We characterized ECM deposition by hyperoxia‐induced senescent fASM via the ECM deposition assay. Hyperoxia‐treated cells showed an increased deposition of COL1 and FN1 compared to normoxia‐exposed cells (Figure 1B). No change was observed in COL3 deposition.

### 3.3 Effects of Fucoidan and D + Q Treatments on Senescence Markers

**FIGURE 2:**
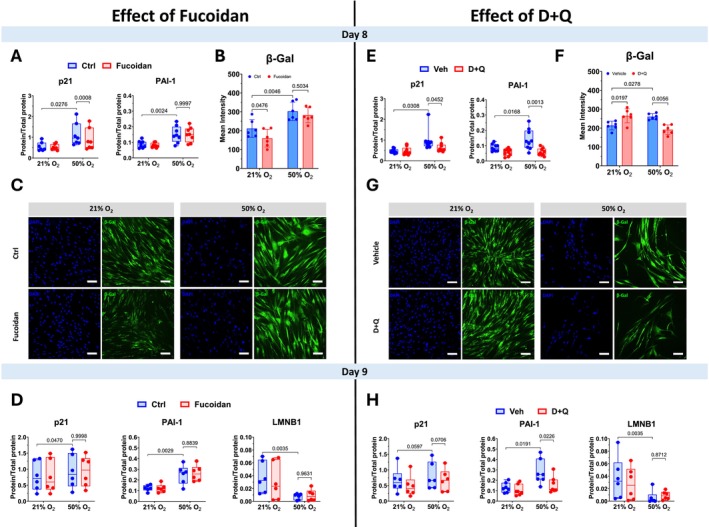
Fucoidan and D + Q treatments mitigate senescence markers in hyperoxia‐induced senescent fASM. Cells were exposed to normoxia (21% O_2_) or hyperoxia (50% O_2_) for 7 days, then treated with Fucoidan or Dasatinib (D) + Quercetin (Q) (D + Q) for 24 h in normoxic environment. Cells and cell lysates were then analyzed for protein expression of senescence marker p21, PAI‐1, LMNB1, and β‐Galactosidase (β‐Gal) activity at day 8 and/or 9. Jess and CellEvent Senescence Green Detection Kit were used to assess these markers. Images were captured using Alexa Fluor 488/FITC filter set, with a Nikon fluorescence microscope and a dry ×20 objective. For each primary cell line, technical duplicates were performed. The mean intensities presented as averages from 527 to 1119 cells per condition. The effects of Fucoidan (A–D) and D + Q (E–H) on senescence markers are presented. For statistical analysis, 2‐way ANOVA with Tukey's multiple comparisons test was applied. Data are presented as box plot or mean ± SD of *n* = 6–8 cell lines per group. *p* value < 0.05 was considered statistically significant. LMNB1, Lamin B1; PAI‐1, Plasminogen activator inhibitor‐1; SD, standard deviation. Scale bar: 100 μM.

Cell lysates following normoxia vs. hyperoxia for 7 days with or without treatment with Fucoidan or D + Q for 24 h were analyzed for protein expression of p21, PAI‐1, and LMNB1 using JESS, and β‐Gal activity using CellEvent Senescence Green Detection Kit at day 8 and 9. Fucoidan reduced p21 but not PAI‐1 expression in hyperoxia‐exposed cells at day 8 (Figure 2A). However, Fucoidan significantly reduced β‐Gal activity in normoxia‐exposed cells, but had no effect on hyperoxia‐exposed cells (Figure 2B,C). An additional analysis on sample from day 9 showed no effect of Fucoidan on expression levels of p21, PAI‐1, and LMNB1 (Figure 2D).

D + Q treatments decreased expression levels of p21, PAI‐1 (Figure 2E), and β‐Gal activity in hyperoxia‐exposed fASM at day 8 (Figure 2F,G). Surprisingly, β‐Gal activity was increased in fASM exposed to normoxia and treated with D + Q. Furthermore, expression levels of p21 and PAI‐1 remained low at day 9 in hyperoxia‐exposed cells treated with D + Q (Figure 2H). No D + Q effect was observed on LMNB1 in normoxia and hyperoxia‐exposed cells after D + Q treatment.

### 3.4 Hyperoxia‐Induced Senescent fASM Secrete Higher Level of SASP Proteins

**FIGURE 3:**
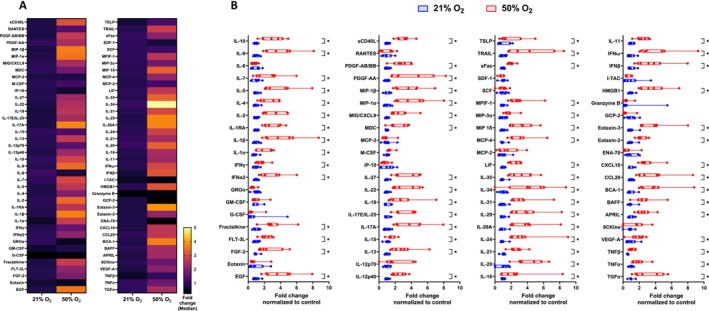
Hyperoxia‐induced senescent fASM secrete higher levels of SASP. Cells cultured till day 7 in normoxic (21% O_2_) or hyperoxic (50% O_2_) environment were incubated with fresh growth media in normoxia for 24 h, and the supernatants were collected. Samples were then analyzed for SASP secretion by Eve Technologies Corporation (Calgary, Alberta, Canada) using Luminex xMAP and the Human Cytokine/Chemokine 96‐Plex Discovery Assay Array (HD96) panel. (A) Out of the 96 proteins, 80 were detected and visualized using a heatmap. (B) A detailed analysis of each detected marker is shown as fold‐change normalized to control. For statistical analysis, 2‐way ANOVA with a two‐stage linear step‐up procedure of Benjamini, Krieger and Yekutieli test, with individual variances computed for each comparison was applied. False discovery rate < 0.05 was considered significant. Data are presented as box plot of *n* = 7 cell lines per group.

Supernatants collected at day 8 from normoxia and hyperoxia‐exposed fASM were analyzed for SASP secretion by Eve Technologies Corporation (Calgary, Alberta, Canada) using the Human Cytokine/Chemokine 96‐Plex Discovery Assay Array (HD96) panel. This analysis examined the secretion of SASP proteins over 24 h (day 7–day 8). Out of 96 proteins analyzed, 80 proteins were detected and visualized in the heatmap (Figure 3A). A total of 62 proteins were significantly increased in supernatants from hyperoxia‐exposed fASM compared to supernatants from normoxia‐exposed fASM (Figure 3B).

### 3.5 Fucoidan Inhibits SASP Secretion by Hyperoxia‐Induced Senescent fASM, but Not D + Q

**FIGURE 4:**
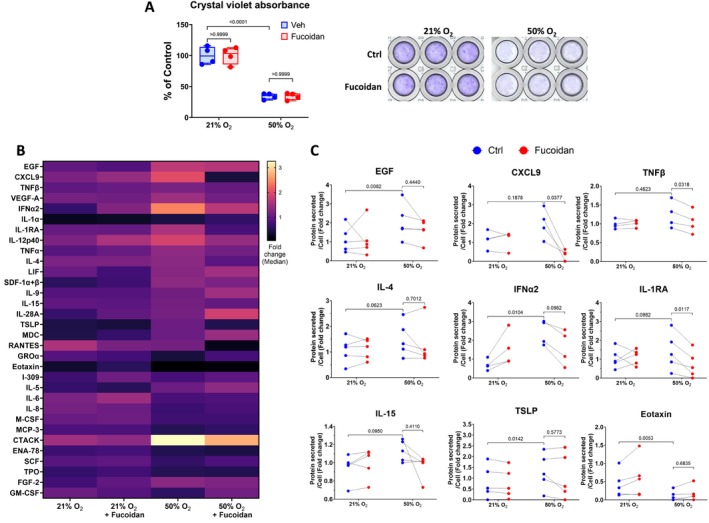
Fucoidan reduced SASP secretion by hyperoxia‐induced senescent fASM. Cells and Supernatants were harvested at day 9 from cells cultured for 7 days in normoxia (21% O_2_) or hyperoxia (50% O_2_), then treated with Fucoidan [100 μg/mL] for 24 h, and subsequently incubated with growth medium for 24 h in normoxia. Cell proliferation and SASP secretion were analyzed using Crystal Violet Staining (A) and multiplex assay (Human Cytokine/Chemokine 71‐Plex Discovery Assay Array (HD71)) (B), respectively. (C) Cytokines and chemokines that were significantly increased in supernatants from hyperoxia‐exposed cells or decreased in supernatants from hyperoxia‐exposed cells treated with Fucoidan are presented. For statistical analysis, 2‐way ANOVA with Tukey's multiple comparisons test was applied. A total of *n* = 4–5 cell lines were analyzed per group. *p* value < 0.05 was considered statistically significant. OD, optical density.

**FIGURE 5:**
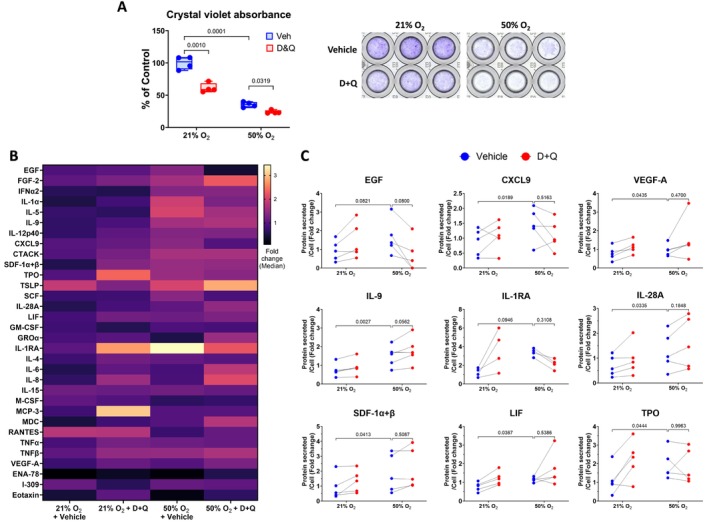
Effect of D + Q on hyperoxia‐induced SASP proteins. Cells and supernatants were harvested at day 9 from cells cultured for 7 days in normoxia (21% O_2_) or hyperoxia (50% O_2_), then treated with 0.05% DMSO (vehicle) or D + Q [250 nM + 375 nM] for 24 h, and subsequently incubated with growth medium for 24 h in normoxia. Cell proliferation and SASP secretion were analyzed using Crystal Violet Staining (A) and multiplex assay (Human Cytokine/Chemokine 71‐Plex Discovery Assay Array (HD71)), respectively. (B) Out of 71 proteins, 32 proteins were detected and are visualized in the heatmap. (C) D + Q did not decrease SASP secretion at day 9 in hyperoxia‐induced senescent cells. For statistical analysis, 2‐way ANOVA with Tukey's multiple comparisons test was applied. A total of *n* = 4–5 cell lines were analyzed per group. *p* value < 0.05 was considered statistically significant. D, Dasatinib; OD, optical density; Q, Quercetin.

To assess cell viability, Crystal Violet staining was performed on adherent cells. Solubilization of Crystal Violet enables quantification of cell number, and absorbance is directly proportional to the number of cells. Absorbance is presented as percentage of control. The cell number was higher for normoxia‐exposed fASM compared to hyperoxia‐exposed fASM (Figures 4A and 5A). However, no difference was observed in Fucoidan‐treated cells, highlighting that Fucoidan does not cause cell death in normoxia or hyperoxia. On the other hand, D + Q‐treated cells (normoxia or hyperoxia‐exposed) showed a significant decrease in cell number. To our surprise, D + Q showed a stronger effect on normoxia‐exposed cells (Figure 5A), suggesting that D + Q does not kill only senescent cells.

Following quantification of senescence markers with Fucoidan or D + Q treatment, the supernatants collected from all treated groups at day 9 were analyzed for SASP secretion using the Human Cytokine/Chemokine 71‐Plex Discovery Assay Array (HD71) panel. A total of 32 proteins were detected and visualized in the heatmaps (Figures 4B and 5B). Detailed analysis showed that only six proteins including epidermal growth factor (EGF), interleukin‐4 (IL‐4), interferon alpha‐2 (IFNα2), interleukin‐1 receptor antagonist (IL1RA), IL‐15 and thymic stromal lymphopoietin (TSLP) were increased or trended towards increase by hyperoxia‐induced senescent cells compared to normoxia‐exposed cells (Figure 4C). Heatmap analysis examined the secretion of SASP proteins over 24 h (day 8–day 9). Fucoidan significantly decreases the secretion of C‐X‐C motif chemokine ligand 9 (CXCL9), IL1RA, and tumor necrosis factor beta (TNFβ) by hyperoxia‐induced senescent cells (Figure 4C).

The hyperoxia‐vehicle samples showed increased levels for seven proteins such as CXCL9, vascular endothelial growth factor A (VEGF‐A), IL‐9, IL‐28A, stromal cell‐derived factors 1‐alpha, and 1‐beta (SDF‐1α + β), leukemia Inhibitory Factor (LIF), and thrombopoietin (TPO) compared to normoxia‐vehicle samples (Figure 5C). However, D + Q‐treatment did not reduce hyperoxia‐increased levels of these proteins.

### 3.6 Hyperoxia Increases a Senescent fASM Cell Cluster That Is Suppressed by Antioxidant MitoQ

**FIGURE 6:**
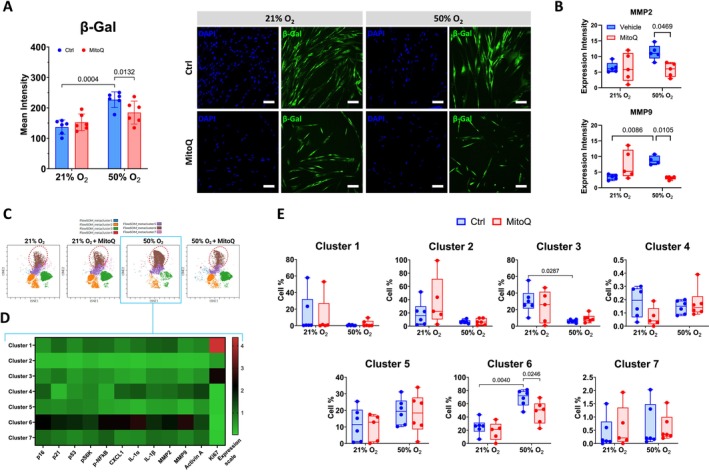
MitoQ inhibits cellular senescence in hyperoxia‐exposed fetal ASM. fASM cells were cultured in normoxia (21% O_2_) or hyperoxia (50% O_2_) for 7 days with vs. without MitoQ [100 nM]. (A) For the detection of senescence‐associated β‐Galactosidase (β‐Gal) the CellEvent Senescence Green Detection Kit was used. Hyperoxia increased β‐Gal intensity, while MitoQ inhibited the increase of β‐Gal intensity in hyperoxia‐exposed cells. Images were captured using Alexa Fluor 488/FITC filter set, with a Nikon fluorescence microscope and a dry ×20 objective. For each cell line, technical duplicates were performed. The mean intensities presented as averages from 402 to 1024 cells per condition. Separately, cells were stained with CyTOF antibodies and run through CyTOF analysis. Acquired data were analyzed using Cytobank software t‐Distributed Stochastic Neighbor Embedding (t‐SNE) and Flow Self‐Organizing Map (FlowSOM) that clustered fASM cells based on their marker expression patterns. (B) The differences in expression intensities of MMP2 and MMP9 in fASM exposed to normoxia or hyperoxia and treated with MitoQ are presented. (C) Seven cell clusters were identified in this experiment. The cluster 6 cell population (circled with red dotted lines) was increased by moderate hyperoxia and decreased by MitoQ treatment. (D) The heatmap shows the expression intensities of each senescence marker by each cell cluster for hyperoxia (50% O_2_) treated fASM. (E) The box plots show the percentage of each cell cluster for each treatment group. For statistical analysis, 2‐way ANOVA or mixed effects analysis with Tukey's multiple comparisons test was applied. Data are presented as mean ± SD or box plot of *n* = 5–6 cell lines per group. *p* value < 0.05 was considered statistically significant. MMP‐2, matrix metalloproteinase‐2; MMP‐9, matrix metalloproteinase‐9; SD, standard deviation. Scale bar: 100 μM.

To investigate senescent cells at the single‐cell level, we first established a panel of CyTOF antibodies to detect senescent cells (Table 1). Using this antibody panel, we examined hyperoxia‐induced senescence in fASM. Our recent study using bulk cell culture showed that moderate hyperoxia increases reactive oxygen species (ROS) production and that MitoQ, a mitochondria‐targeted antioxidant, inhibits hyperoxia‐induced p21 and Bcl‐xL expression in fASM cells (Koloko Ngassie et al. 2025). To delve deeper into these hyperoxia and MitoQ effects at the single‐cell level, we exposed fASM cells to normoxia or hyperoxia with or without MitoQ for 7 days. Hyperoxia‐exposed fASM treated with MitoQ showed lower β‐Gal activity, but not the normoxia‐exposed fASM (Figure 6A). In parallel, cells were processed for CyTOF and data analyzed with t‐SNE and FlowSOM clustering algorithm. The analysis revealed seven distinct cell clusters (Figure 6C), each representing a group of cells with similar phenotypic characteristics. Notably, the heatmap showed that cells in cluster 6 expressed high levels of p16, p21, p53, PS6K, p‐NF‐κB p65, CXCL1, IL‐1α, IL‐1β, MMP2, MMP9, Activin A, and low levels of the proliferation marker Ki67 (Figure 6D), indicating this cluster as senescent cells. A separate analysis of MMP2 and MMP9 expression intensities showed that hyperoxia increases MMP9 level. MitoQ decreases the levels of MMP2 and MMP9 in hyperoxia exposed fASM (Figure 6B). Compared to normoxia group, hyperoxia‐exposed fASM had significantly higher percentages of cluster 6 cells which were lowered by MitoQ treatment (Figure 6E). Hyperoxia also decreased cluster 3 cells that express high levels of Ki67, but MitoQ did not have significant effects on this cluster. Hyperoxia did not significantly change the other five cell clusters.

Hyperoxia‐induced oxidative stress in fASM is not limited to mitochondria but also involves other compartments including the cytosol, as our previous study showed (Koloko Ngassie et al. 2025). We tested catalase, an enzyme targeting cytosolic ROS, but did not find any significant effect on p21 expression in hyperoxia‐exposed fASM (Supporting Information S1, Figure S5B). Hyperoxia can cause mitochondria fragmentation; therefore, we examined the effects of Fucoidan, D + Q, and MitoQ on mitochondria dynamic (Supporting Information S1, Figure S5A). None of them showed an inhibitory effect on mitochondria fragmentation in hyperoxia‐exposed fASM.

## 4 Discussion

Moderate hyperoxia (< 60% O_2_) in premature infants is beneficial initially but detrimentally affects bronchial airways in the long term leading to airway obstruction and AHR (Denis et al. 2001). The underlying mechanisms for hyperoxia‐induced airway pathology are not fully understood. Our previous studies suggest that moderate hyperoxia induces cellular senescence in developing ASM (Koloko Ngassie et al. 2025; Parikh, Britt Jr., et al. 2019). Recognizing that senescence can be detrimental (but also beneficial), in the current study, we examined several approaches to inhibit or prevent the harmful effects of moderate hyperoxia on fASM via modulation of senescence. Here, we explored the effects of Fucoidan, D + Q or MitoQ in alleviating cellular senescence. Our results showed that moderate hyperoxia increases β‐Gal activity, ECM deposition, p21, and PAI‐1, and decreases LMNB1, establishing the link between oxygen and senescence. Hyperoxia‐exposed cells treated with Fucoidan showed a decrease in p21 expression and inhibition of SASP release without inducing cell death. In contrast, hyperoxia‐exposed cells treated with D + Q exhibited decreased β‐Gal activity, p21 and PAI‐1 expression levels, lower cell number, but had no change in SASP. Lastly, hyperoxia‐exposed cells treated with MitoQ showed lower levels of senescence markers and SASP, and lower percentages of senescent cells. These results suggest that Fucoidan and D + Q act as senomorphic and senolytic, respectively, while MitoQ has a prophylactic effect against moderate hyperoxia in developing airway cells.

Extensive studies have shown that senescence plays an important role in various normal physiological as well as pathological processes. Physiologically, senescence participates in embryonic tissue development, tissue repair, and wound healing. Pathologically, senescence contributes to aging and age‐associated diseases. Despite considerable progress in the field, identifying senescent cells remains a significant challenge, as there is currently no consensus on definitive markers. Therefore, the Senescence Network (SenNet) recommends the use of multiple hallmarks including markers linked to cell cycle arrest, activation of the DNA damage response, nuclear and cell morphology changes, upregulation of anti‐apoptotic factors, increased SASP and lysosomal content, and metabolic adaptations (Suryadevara et al. 2024). We previously showed that moderate hyperoxia induces cellular senescence in fASM with increases in markers such as p21, PAI‐1, Bcl‐xL, Cav‐1, and β‐Gal activity, and decreased Ki67, LMNB1, and Caspase‐3 following 7 days of hyperoxia (Koloko Ngassie et al. 2025; Parikh, Britt Jr., et al. 2019). In a previous study, we examined β‐Gal activity at day 7, while the current study assessed the β‐Gal activity over time (day 0, 1, 3, 7, and 8). β‐Gal activity significantly increased in a time‐dependent manner with hyperoxia. Surprisingly, normoxia‐exposed fASM also showed a time‐dependent increased β‐Gal activity. Dimri and colleagues previously showed an increased β‐Gal activity in immortalized fetal lung fibroblast (WI‐38) which became quiescent after reaching 100% confluency (Dimri et al. 1995). Subsequently, reseeding these cells led to loss of positive β‐Gal staining within 2 days. Normoxia‐exposed fASM showed a higher confluency compared to hyperoxia‐exposed cells, indicating that the increased β‐Gal activity in normoxia‐exposed fASM may be linked to cell confluency rather than a reflection of increased senescence.

A key strength of our study is the use of CyTOF single‐cell profiling, which enabled the simultaneous identification of multiple markers within individual cells. This high‐dimensional analysis revealed a specific cell population (cluster 6) co‐expressing high levels of p16, p21, p53, and SASP components, alongside low levels of the proliferation marker Ki67 (Figure 6D). The detection of this cluster, possessing all the key markers of senescence, strengthens the conclusion that these are bona fide senescent cells. Importantly, the percentage of cluster 6 cells significantly increased after hyperoxia exposure and decreased with MitoQ treatment. Cluster 3, which showed opposite expression of senescence markers that were analyzed, was significantly decreased upon hyperoxia exposure. This cluster may represent proliferating cells and show reduced proliferation following hyperoxia. However, MitoQ treatment did not prevent the decrease of this cell population, suggesting a more targeted effect on senescence per se. This single‐cell data corroborates our previous findings from bulk cell analysis, confirming that moderate hyperoxia induces a genuine senescent state in fASM (Koloko Ngassie et al. 2025), and we now show the beneficial effect of approaches such as MitoQ.

Our study is the first to characterize a large group of SASP proteins in the context of hyperoxia‐induced senescence in developing airway cells. These results give us an overview of the pro‐inflammatory molecules that may contribute to airway inflammation and remodeling, and AHR in the perinatal period. For example, IL‐1β, IL‐4, IL‐5, IL‐10, IL‐13, and TSLP, which were highly secreted by hyperoxia‐exposed fASM, are known for their important role in mediating AHR (Bossé 2012; Bradding et al. 2024). The high expression levels of IL‐1α and IL‐1β in hyperoxia‐exposed fASM from the CyTOF analysis correlate with their secretory profile in the supernatants of hyperoxia‐induced senescent fASM, validating our previous findings in fASM exposed to 40% O_2_ (Parikh, Britt Jr., et al. 2019). Additionally, we also showed similar changes in secreted levels of IL‐1α and IL‐1β in fetal human lung fibroblasts (You et al. 2019). In contrast to hyperoxia (40% O_2_) exposed fetal human lung fibroblasts showing no changes in COL1 and FN1 deposition (You et al. 2019), fASM exposed to 50% O_2_ showed an increased deposition of COL1 and FN1. Exposure of naïve fASM to conditioned media from hyperoxia (40% O_2_) exposed fASM also resulted in significant deposition of COL1 and FN1 (Parikh, Britt Jr., et al. 2019). SASP proteins inducing the production of ECM components such as platelet‐derived growth factor (PDGF), epidermal growth factor (EGF), IL‐1β, IL‐13, and tumor necrosis factor alpha (TNFα) (Giblin et al. 2022; Sutherland et al. 2023) were highly secreted by hyperoxia‐induced senescent fASM. Hyperoxia‐driven increased level of MMP9, which is known to degrade COL1, may contribute to remodeling of ECM. The inhibition of MMP2 and MMP9 by MitoQ highlights the crucial role of mitochondrial ROS in ECM remodeling. Although the activity of the enzymes MMP2 and MMP9 were themselves not assessed in this study, our previous study showed an increased activity of MMP9 in fASM upon hyperoxia exposure (Vogel et al. 2017). Our data and previous studies suggest a cell‐ and perhaps oxygen level‐dependent response to hyperoxia and a role for ECM/modifier SASP proteins that could regulate airway remodeling via autocrine and paracrine signaling.

Given potential detrimental effects of accumulated senescent cells in tissues/organs, targeting these cells has become one of the therapeutic approaches against age‐related diseases. Senescent cells and their SASPs can be targeted using senolytics and senomorphics. However, and understandably, there has not been much focus on using such approaches in the perinatal period, given that senescence has beneficial effects during organ development, and furthermore targeting of detrimental senescence per se requires a high threshold of safety and efficacy in vulnerable populations such as preterm infants. Nonetheless, our data show that senescence‐focused therapies might be effective in developing lung cells. Here, Fucoidan is an interesting therapy. Fucoidan showed an anti‐inflammatory effect, without killing cells or diminishing the levels of some senescence markers. Fucoidan has shown its anti‐inflammatory potential in several inflammatory diseases including cancer, neurodegenerative diseases, cardiovascular diseases and diabetes (Sanjeewa et al. 2021) and in an in vitro study (Ahmad et al. 2021). Our single‐cell analysis showed increased p‐NF‐κB‐positive fASM upon hyperoxia exposure. Since Fucoidan achieves its anti‐inflammatory mechanism via inhibition of pro‐inflammatory signaling pathways such as NF‐κB and mitogen‐activated protein kinases (MAPKs) (Sanjeewa et al. 2021), we can theorize that Fucoidan decreases SASP secretion in fASM via inhibition of NF‐κB and/or MAPKs pathways. Further studies are needed to identify by which mechanism Fucoidan inhibits the expression of proinflammatory molecules. Overall, our findings suggest that Fucoidan may act as a senomorphic and could be used to manage hyperoxia‐induced SASP release.

Emerging evidence suggests that targeting senescent cells with senolytics alleviates the detrimental effects of senescence and improves healthspan across various biological systems and pre‐clinical models (Suda et al. 2025). Among senolytics, the D + Q combination has proven to be the most effective to date. D + Q shows efficacy in eliminating senescent cells, delaying aging, and mitigating disease progression in models of dementias (Krzystyniak et al. 2022; Zhang et al. 2019), idiopathic pulmonary fibrosis (Schafer et al. 2017), cardiovascular diseases, and frailty (Zhu et al. 2015). Our previous and current cell analysis revealed that D + Q treatment reduces hyperoxia‐induced cellular senescence markers including colocalization of γ‐H2AX with p16/p21 (Parikh, Britt Jr., et al. 2019), p21, PAI‐1 levels, and β‐Gal activity in fASM. However, in our study, surprisingly, no effect was observed on SASP levels. A previous study by Maurer and colleagues showed a reduction in IL‐6 and CXCL1 release by D + Q treated human articular chondrocytes. Here the cells were treated for 3 days with D + Q, then treatment was replaced with growth medium and cultured for an additional 4 days without D + Q before the supernatants were analyzed for SASP secretion (Maurer et al. 2025). This suggests that an inhibitory effect of D + Q on SASP release could be observed in our in vitro model after an extended incubation period after D + Q removal. Further experiments will be needed to test this hypothesis. Unexpectedly, D + Q reduced cell number in normoxia‐exposed cells, but did not reduce the levels of senescence markers. A recent study performed on vascular smooth muscle cells from young human (29–34 years) showed that D + Q [200 nM + 5 μM] treatment affects their chromatin structure and negatively impacts their proliferation (Gadecka et al. 2025). This highlights a potential cytotoxic effect of D + Q on naïve cells, and emphasizes the importance of drug dosage as well as assessment of its use based on context.

While the use of senomorphics or senolytics involves the alleviation of established senescence following hyperoxia exposure, there has consistently been an interest in preventing hyperoxia effects with the recognition that oxygen induces oxidant stress and influences mitochondria. Accordingly, we explored whether detrimental effects of hyperoxia could be prevented using the mitochondrial‐targeted ROS inhibitor MitoQ. We previously showed that hyperoxia increases mitochondrial ROS in fASM and that the use of MitoQ inhibits the expression of hyperoxia‐induced senescence markers p21 and Bcl‐xL (Koloko Ngassie et al. 2025). Our current single‐cell analysis reveals that moderate hyperoxia specifically increases the numbers of senescent cluster 6 cells and this increase is inhibited by MitoQ treatment. Previous studies highlighted the positive effects of MitoQ on senescence markers. MitoQ prevented cigarette smoke extract (CSE) induced cellular senescence by lowering the levels of β‐Gal activity, p16, p21, and γ‐H2AX in mouse alveolar type 2 cells (Zhang et al. 2021). Additionally, Abdeahad and colleagues showed that Doxorubicin‐induced cellular senescence in human umbilical vein endothelial cells (HUVECs) was prevented via MitoQ treatment which decreases the gene expression of senescence markers (*CDKN2A/p16*, *CDKN1A/p21*, and *p53*), SASP markers and decreased β‐Gal positive cells (Abdeahad et al. 2025). These findings are in line with our results showing the inhibitory effect of MitoQ on the expression levels of senescence and SASP markers in hyperoxia‐exposed fASM.

In summary, our study reveals that moderate hyperoxia‐induced senescent fASM cells are a druggable target. We demonstrate distinct, successful strategies using the senomorphic Fucoidan, the senolytic D + Q, and the prophylactic antioxidant MitoQ. These findings lay a foundation for future in vitro and in vivo studies examining the role of senotherapeutics and the protective role of mitochondrial‐targeted antioxidants in the context of hyperoxia‐induced senescence in preterm infants. Considering that cellular senescence contributes to embryonic tissue development (Muñoz‐Espín et al. 2013; Storer et al. 2013) and normal postnatal lung development (Yao et al. 2023), it is critical for future studies to determine how best to selectively target the detrimental senescent fASM cells without compromising the beneficial ones. Future work should explore optimizing treatment dosages and timing or a potential combination strategy, such as prophylactic MitoQ followed by a low‐dose senolytic. Developing such precise interventions is a promising path towards mitigating hyperoxia‐driven airway inflammation, AHR, and remodeling in preterm infants.

## Author Contributions

Maunick Lefin Koloko Ngassie, Li Y. Drake, Christina M. Pabelick, and Y.S. Prakash conceived and designed research; Maunick Lefin Koloko Ngassie, Li Y. Drake, Yi Zhu, Yamillie Ortiz, Daniel A. Pfeffer‐Kleemann, Michael A. Thompson, and Samantha K. Hamrick performed experiments; Maunick Lefin Koloko Ngassie, Li Y. Drake, Yamillie Ortiz, and Samantha K. Hamrick prepared figures; Maunick Lefin Koloko Ngassie, Li Y. Drake, Yi Zhu, Yamillie Ortiz, and Samantha K. Hamrick analyzed data; Maunick Lefin Koloko Ngassie, Li Y. Drake, Yi Zhu, Christina M. Pabelick, and Y.S. Prakash interpreted results of experiments; Maunick Lefin Koloko Ngassie and Li Y. Drake drafted manuscript; Maunick Lefin Koloko Ngassie, Li Y. Drake, Yi Zhu, and Y.S. Prakash edited and revised manuscript; Maunick Lefin Koloko Ngassie, Li Y. Drake, Yi Zhu, Yamillie Ortiz, Daniel A. Pfeffer‐Kleemann, Michael A. Thompson, Samantha K. Hamrick, Christina M. Pabelick, and Y.S. Prakash approved final version of manuscript.

## Funding

This study was supported by NIH grants R01 HL158532 (Prakash), R01 HL056470 (Prakash), R01 HL171915 (Pabelick), and R01 AG087387 (Zhu).

## Conflicts of Interest

The authors declare no conflicts of interest.

## Supporting information


**Data S1:** fASM characterization, experimental design, optimization of Fucoidan concentration, mitochondria dynamic, and effect of catalase on p21.

**Data S2:** Representative digital blots from the capillary JESS showing the specificity of the used antibodies targeting the selected senescence markers and total protein.

## Data Availability

The data that support the findings of this study are available on request from the corresponding author. The data are not publicly available due to privacy or ethical restrictions.
