# Supplementary material for: Targeting Hyperoxia‐Induced Cellular Senescence in Developing Human Airway Cells: Senomorphics Versus Senolytics Versus Antioxidants
Source: Aging Cell. 2026 May 8;25(5):e70538. doi: 10.1111/acel.70538 (PMC13154765; doi:10.1111/acel.70538)
Supplement: Supplementary file 1 — Data S1: fASM characterization, experimental design, optimization of Fucoidan concentration, mitochondria dynamic, and effect of catalase on p21. [file ACEL-25-e70538-s002.pdf]

Targeting Hyperoxia-Induced Cellular Senescence In Developing Human Airway Cells: Senomorphics vs. Senolytics vs. Antioxidants

Authors and affiliations: Maunick L. Koloko Ngassie[1], Li Y. Drake[1], Yi Zhu[2], Yamillie Ortiz[1], Daniel A. Pfeffer-Kleemann[1], Michael A. Thompson[1], Samantha K. Hamrick[1], Christina M. Pabelick[1,2], and Y.S. Prakash[1,2*]

[1]Department of Anesthesiology and Perioperative Medicine, Rochester, MN, USA.

[2]Department of Physiology and Biomedical Engineering, Mayo Clinic, Rochester, MN, USA.

Supplemental file 1: fASM characterization, experimental design, optimization of Fucoidan concentration, mitochondria dynamic, and effect of catalase on p21

Immunofluorescence staining for fibroblast specific protein and smooth muscle myosin in fASM

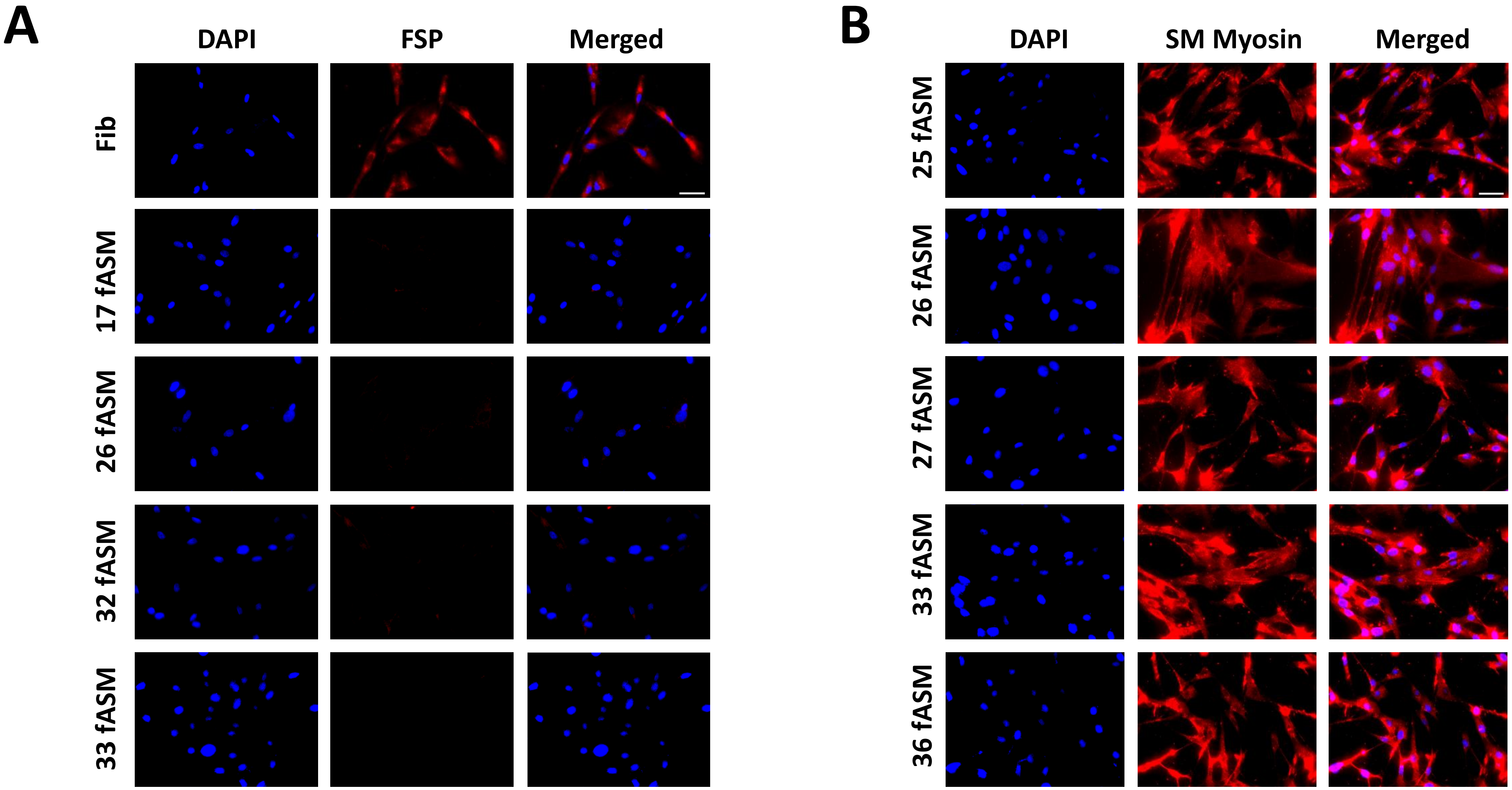

Figure S1: Immunofluorescence staining for fibroblast specific protein (FSP) and smooth muscle (SM) Myosin expression in fetal airway smooth muscle cells (fASM). fASM were fixed and processed for immunofluorescent staining of **A**) FSP and **B**) SM Myosin. Human lung fibroblasts (Fib) were used as a positive control for FSP staining. Images were captured using a Nikon Eclipse Ti imaging system. Staining of 4-5 biological replicates were performed. Scale bar = 50µm

Calcium response of fASM upon Acetylcholine or Histamine exposure

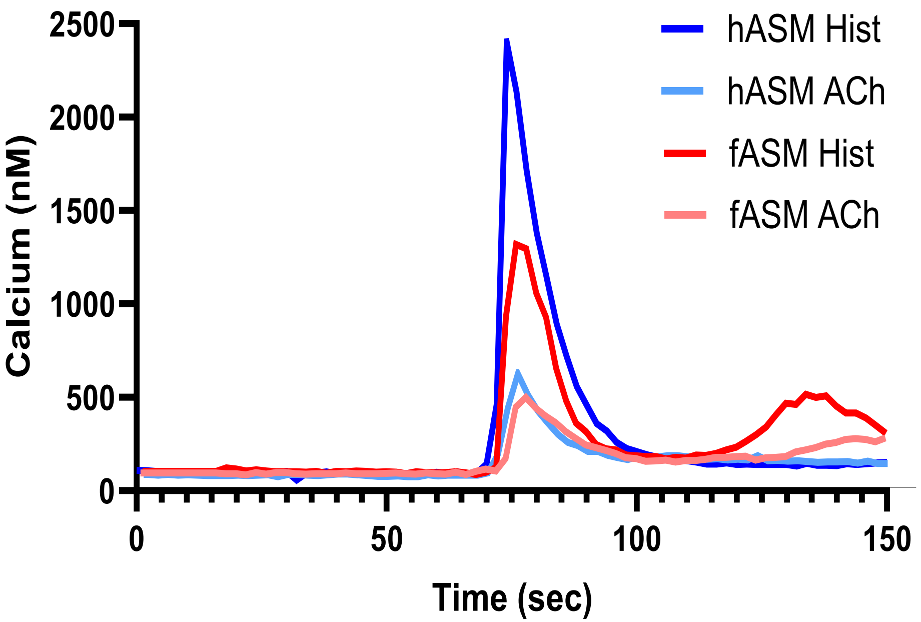

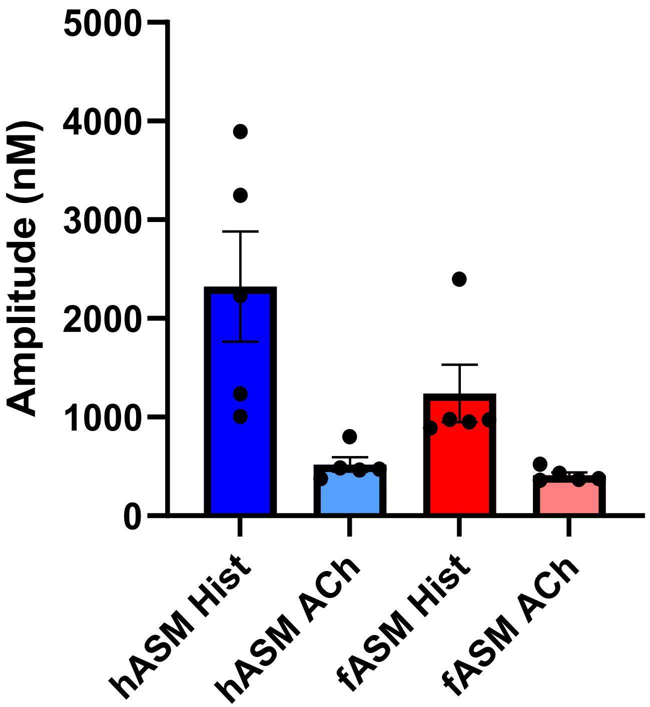

Figure S2: [Ca^{2+}] responses to agonists in fetal airway smooth muscle cells (fASM) and adult human airway smooth muscle cells (hASM). fASM and hASM were serum deprived for 24 hours then loaded with Fura-2/AM for live-cell fluorescent imaging of [Ca^{2+}] response to 10 µM Acetylcholine (Ach) and Histamine (His). Amplitude was calculated between background-adjusted baseline to maximum peak $[Ca^{2+}]_m$. Representative tracings (one of five) are shown. 5 biological replicates were performed.

Experimental design

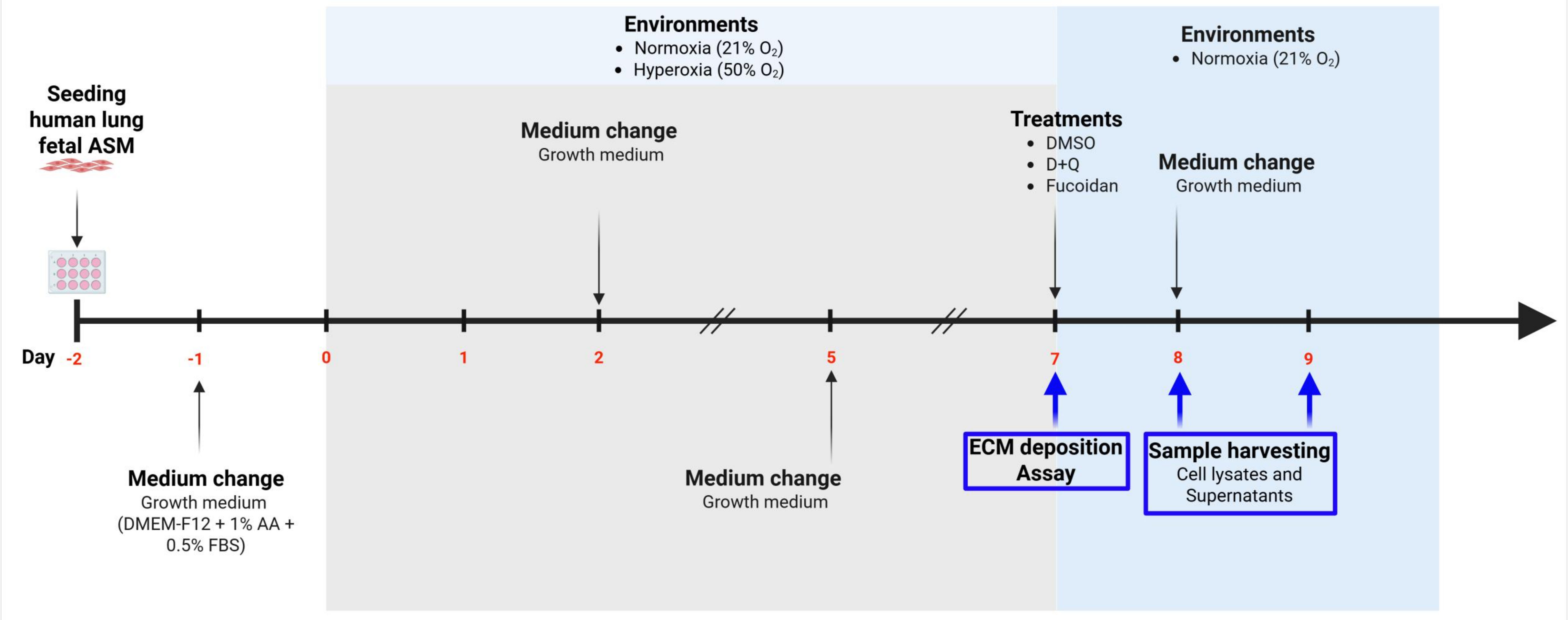

Figure S3: Experimental design for cell culture and senotherapeutics treatments. Fetal airway smooth muscle cells (fASM) were plated, made quiescent and cultured for 7 days in normoxic or hyperoxic environment. The cells were treated with vehicle (0.05% DMSO), D+Q [250 nM + 375 nM] or Fucoidan [100 µg/mL] in normoxic environment for 24h, then treatments were replaced with growth medium and incubated for additional 24h. Plate for ECM deposition assay were harvested at day 7, cell lysates and supernatant were harvested at day 8 and 9. BioRender was used to create this figure. DMEM-F12, Dulbecco's Modified Eagle's Medium/Ham's Nutrient Mixture F 12 ; AA, antibiotics and antimycotic; FBS, fetal bovine serum; D, Dasatinib; Q, Quercetin

Optimization of Fucoidan concentration

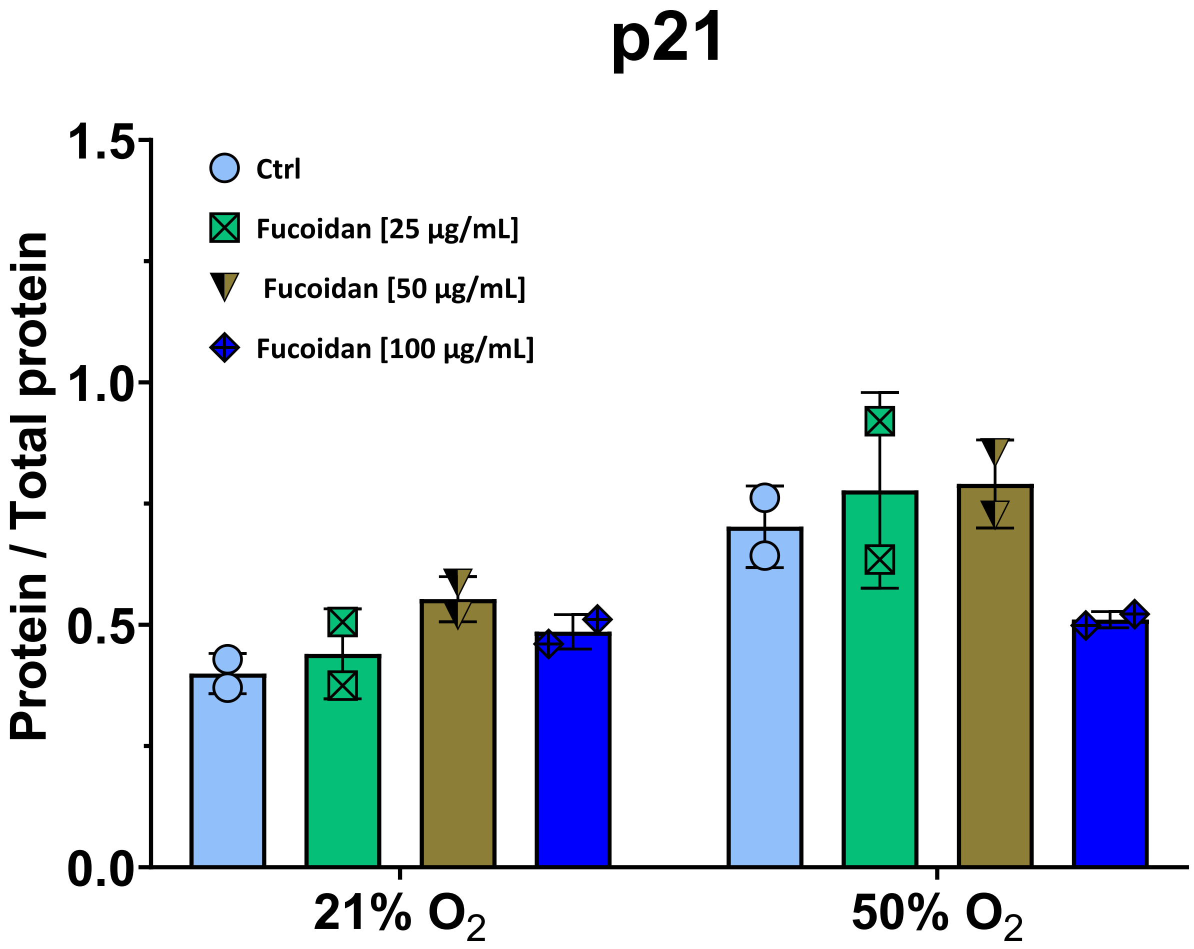

Figure S4: Determining the optimal concentration of Fucoidan for the treatment of hyperoxia-induced senescent fetal airway smooth muscle cells (fASM). fASM) were plated, made quiescent (for 24h) and cultured for 7 days in normoxia or hyperoxia. Then, cells were treated with Fucoidan ([25 µg/mL], [50 µg/mL] and [100 µg/mL]) for 24h in normoxic environment, and cell lysates were harvested and analyzed using JESS for p21 expression. A concentration of Fucoidan [100 µg/mL] was chosen for further experiments.

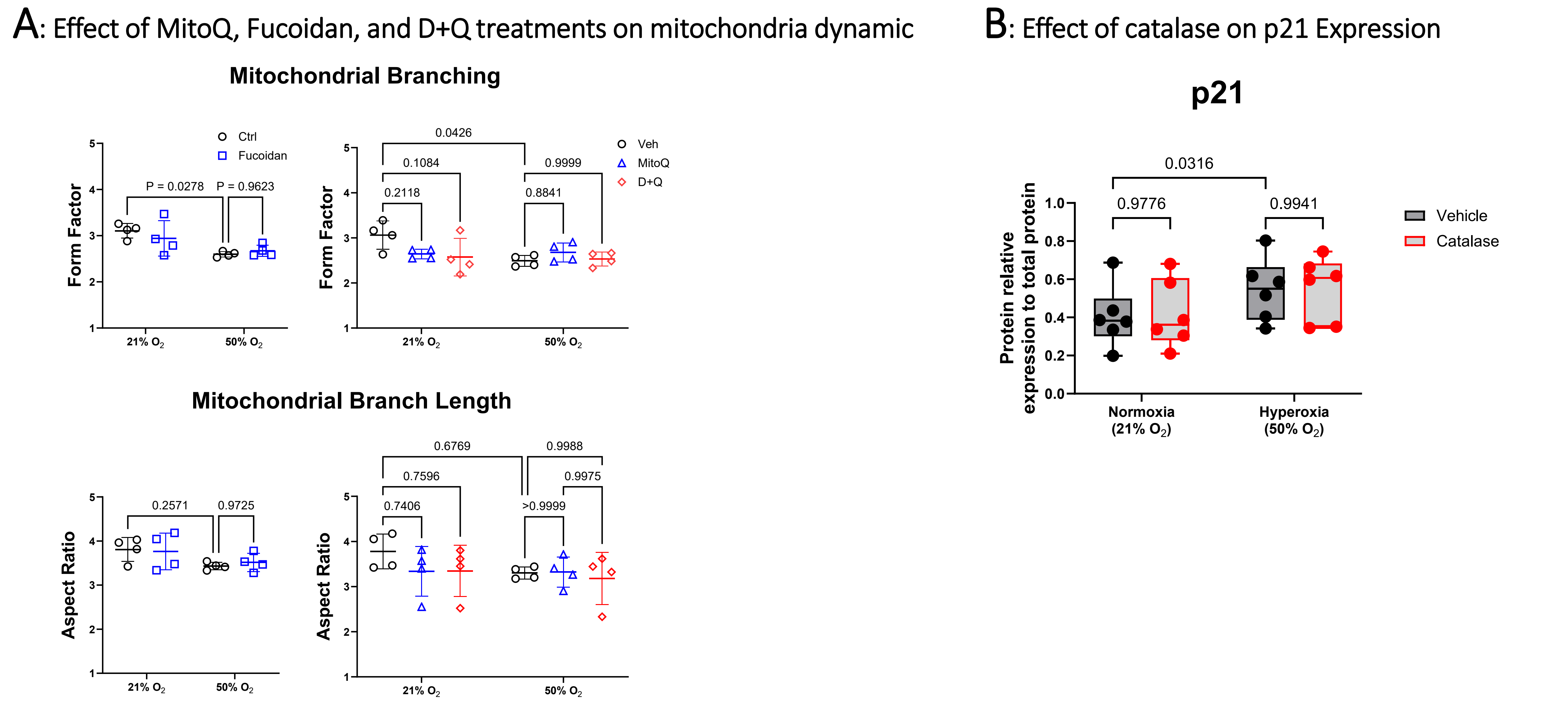

Figure S5: MitoQ, Fucoidan and D+Q treatments have no effect on mitochondria dynamic, and catalase has no effect on p21 expression. **A**) Fetal airway smooth muscle cells (fASM) were exposed to 21% or 50% O_2 and treated with fucoidan [100ug/mL]), MitoQ [100nM], or D+Q [250nM + 375nM]. Mitochondrial branching (form factor) and branch length (aspect ratio) was examined via Mitotracker staining and 'Mitomorph' macro and ImageJ software. The detailed protocol about the calculation of form factor is described in our previous publication (**B**) fASM were exposed to 21% or 50% $O_{2,}$ treated with catalase, and processed for JESS analysis of p21 expression. For statistical analysis a 2-way ANOVA with Tukey's multiple comparison test was applied. p-value < 0.05 was considered statistically significant. Data represent 4-6 biological replicates.
