# Supplementary material for: Targeting Hyperoxia‐Induced Cellular Senescence in Developing Human Airway Cells: Senomorphics Versus Senolytics Versus Antioxidants
Source: Aging Cell. 2026 May 8;25(5):e70538. doi: 10.1111/acel.70538 (PMC13154765; doi:10.1111/acel.70538)
Supplement: Supplementary file 2 — Data S2: Representative digital blots from the capillary JESS showing the specificity of the used antibodies targeting the selected senescence markers and total protein. [file ACEL-25-e70538-s001.pdf]

# Targeting Hyperoxia-Induced Cellular Senescence In Developing Human Airway Cells: Senomorphics vs. Senolytics vs. Antioxidants

**Authors and affiliations:** Maunick L. Koloko Ngassie[1], Li Y. Drake[1], Yi Zhu[2], Yamillie Ortiz[1], Daniel A. Pfeffer-Kleemann[1], Michael A. Thompson[1], Samantha K. Hamrick[1], Christina M. Pabelick[1,2], and Y.S. Prakash[1,2*]

[1]Department of Anesthesiology and Perioperative Medicine, Rochester, MN, USA.

[2]Department of Physiology and Biomedical Engineering, Mayo Clinic, Rochester, MN, USA.

**Supplemental file 2:** Representative digital blots from the capillary JESS showing the specificity of the used antibodies targeting the selected senescence markers and total protein.

# Day 8: representative digital blots of senescence markers and total protein labelling

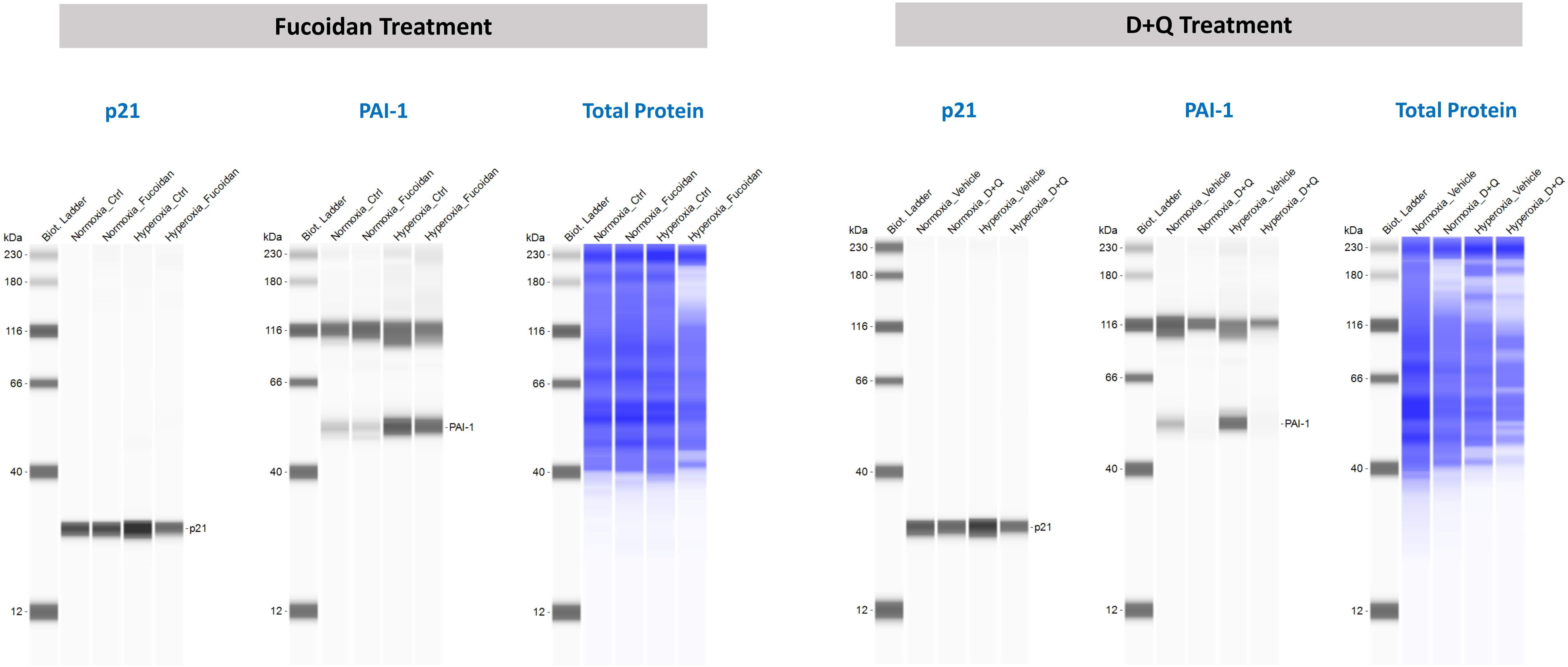


**Figure S1:** Representative digital blots from the capillary JESS showing the specificity of the used antibodies targeting the selected markers of cellular senescence at day 8. Fetal airway smooth muscle cells were plated, made quiescent (for 24h) and cultured for 7 days in normoxia or hyperoxia. Cells were treated with vehicle (0.05% DMSO), D+Q [250 nM + 375 nM] or Fucoidan [100 µg/mL] in normoxic environment for 24h, and cell lysates were harvested (day 8) and analyzed using JESS for the expression of senescence markers: p21 and PAI-1. Total protein labelling was used for the normalization of protein expression. Plasminogen activator inhibitor-1, PAI-1.

# Day 9: representative digital blots of senescence markers

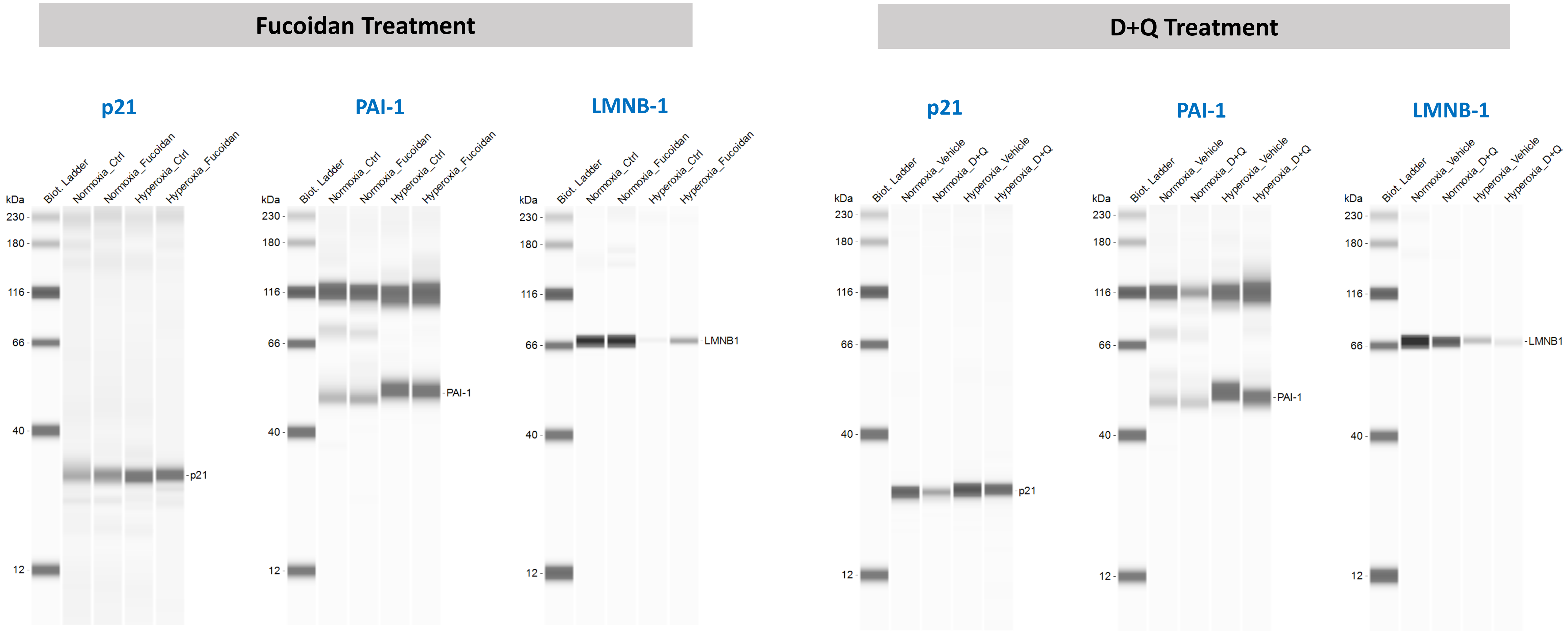


**Figure S2:** Representative digital blots from the capillary JESS showing the specificity of the used antibodies targeting the selected markers of cellular senescence at day 9. Fetal airway smooth muscle cells were plated, made quiescent (for 24h) and cultured for 7 days in normoxia or hyperoxia. Cells were treated with vehicle (0.05% DMSO), D+Q [250 nM + 375 nM] or Fucoidan [100 µg/mL] in normoxic environment for 24h, then treatments were replaced with growth medium and incubated for additional 24h. Cell lysates were harvested and analyzed using JESS for the expression of senescence markers: p21, PAI-1 and LMNB1 . Plasminogen activator inhibitor-1, PAI-1; Lamin B1, LMNB1

# Day 9: representative digital blots of total protein labelling

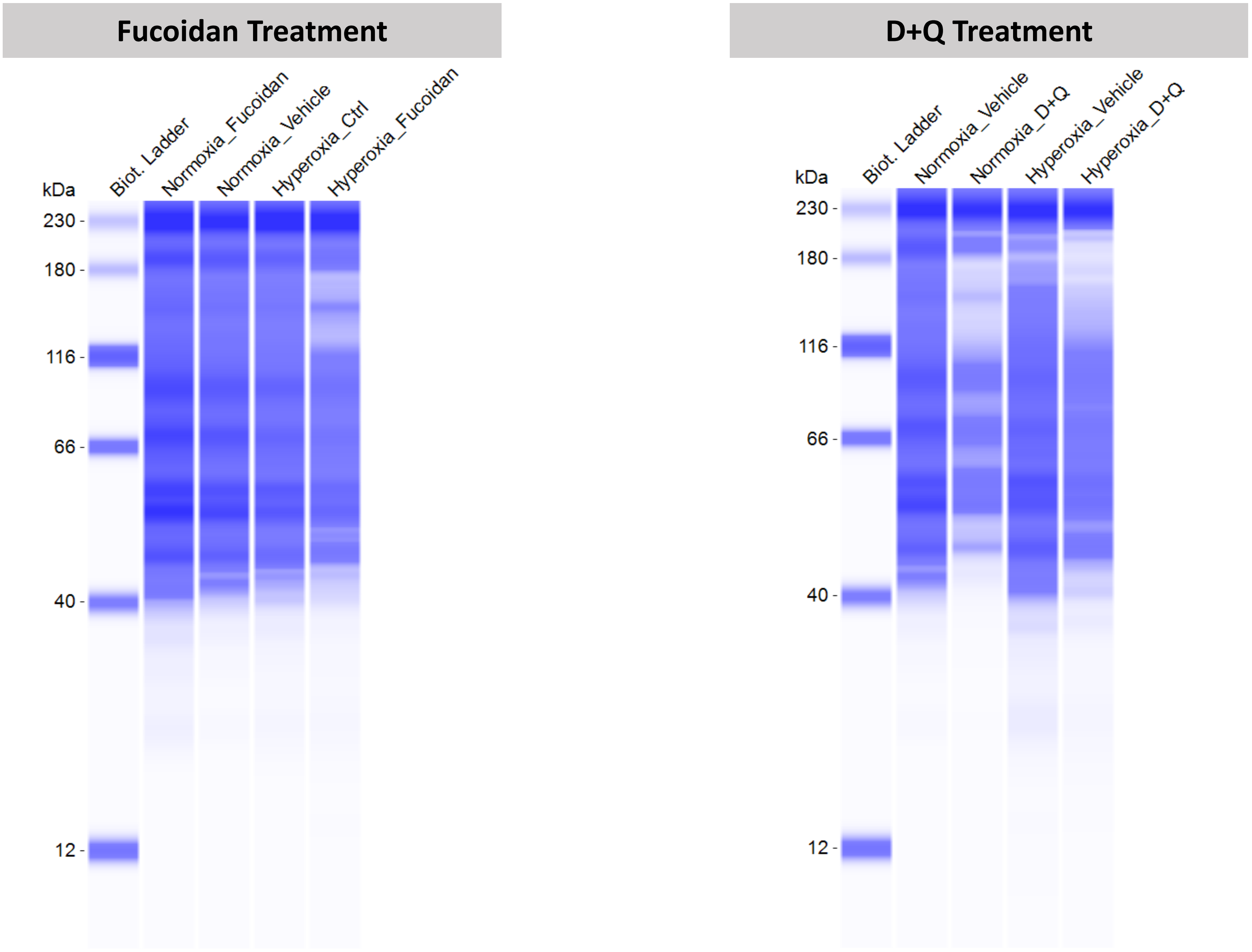


**Figure S3:** Representative digital blots from the capillary JESS showing the total protein labelling used for normalization of protein expression at day 9.
